# The Portuguese observatory on occupational psychosocial factors: contribution for public health: Claudia Fernandes

**DOI:** 10.1093/eurpub/ckac130.196

**Published:** 2022-10-25

**Authors:** C Fernandes, T Cotrim, A Pereira, CF Silva, P Bem-Haja, R Azevedo, S Antunes, J Sousa Pinto, I Silva

**Affiliations:** 1 CATIM, Technological Center for the Metalworking, Porto, Portugal; 2 Ergonomics Laboratory, Faculdade de Motricidade Humana, Universidade de Lisboa, Cruz Quebrada, Portugal; 3 CIAUD, Faculty of Arquitetura, Universidade de Lisboa, Alto da Ajuda, Portugal; 4 CIDTFF, Department of Education and Psychology, University Aveiro, Aveiro, Portugal; 5 WJCR, Department of Education and Psychology, University Aveiro, Aveiro, Portugal; 6 CINTESIS, Department of Education and Psychology, University Aveiro, Aveiro, Portugal; 7 UNICES, University of Maia, Maia, Portugal; 8 Center ALGORITMI, University of Minho, Braga, Portugal; 9 APPsyCI, ISPA, Instituto Universitário, Lisbon, Portugal; 10 IETA, University of Aveiro, Aveiro, Portugal; 11 CICS.NOVA.UMinho, Escola de Psicologia, Universidade do Minho, Braga, Portugal

## Abstract

To achieve the goal of sustainable employment, considering the profile of the Portuguese working population (PWP), is needed a range of strategies to ensure long, productive, and sustainable careers allied with a better quality of working life, health, and wellbeing, but also with public health policies grounded on scientifically validated and reliable data. This is possible through a comprehensive working system approach that ensures workers will be mentally and physically able to remain at work by the balance between work demands and individual resources allied with public health policies transfer into the workplaces by organizations’ leadership and policy makers. The Portuguese Observatory on Occupational Factors (Popsy@Work) aims at addressing this global challenge by: i) digitally collecting psychosocial data on the PWP; ii) implementing and strengthening of a psychosocial occupational health surveillance digital system; iii) providing reference values for the PWP concerning Psychosocial Health; iv) Transferring to society knowledge and best practices; v) Raising awareness on the importance of psychosocial management in occupational settings based on science. Popsy@work is a digital platform that collects and aggregates psychosocial data analytically and creates a visualization hub adding value to data on the PWP and giving science back to society in a usable way, empowering workers, strengthening organizations and grounding public policies. Pospy@Work considers the development of strategic intelligence on levels and inequalities of psychosocial health and well-being in occupational settings by robust metrics and reference data. Creating opportunities for national policy dialogue on inequalities, including the psychosocial health of the PWP through collaboration with diverse sectors identifying and mapping subgroups of populations whose unmet needs require specific outreach measures.

**Key messages:**

- Popsy@work allows psychosocial health monitoring, reporting and foresight, promoting the provision of robust metrics and reference values for the Portuguese working population.
- Popsy@work allows psychosocial health on occupational settings to be digitally monitored in a continuous way and sustain public health policies.

